# Monkeypox-Related Ocular Disease: Disciform Keratitis and Recurrent Keratouveitis in an Immunocompetent Patient in a Resource-Limited Setting

**DOI:** 10.7759/cureus.94933

**Published:** 2025-10-19

**Authors:** Christian García-Mera, Daniela Yosa

**Affiliations:** 1 Emergency Department, Instituto del Cáncer SOLCA Cuenca, Cuenca, ECU; 2 Ophthalmology Department, Clínica Oftalmolaser, Cuenca, ECU

**Keywords:** disciform keratitis, keratouveitis, monkeypox, monkeypox-related ocular disease, ophthalmic topical insulin

## Abstract

Monkeypox-related ocular disease (MPXROD) is an uncommon but potentially sight-threatening complication of monkeypox virus (MPXV) infection. While most cases improve with supportive care, some patients may develop ocular involvement. We describe a 28-year-old immunocompetent man who experienced decreased vision and paracentral dendritic corneal ulcers during the acute phase of infection. Initial treatment, directed at presumed herpetic keratitis, successfully resolved the ulcers but left a residual stromal leukoma. Subsequent inflammatory flares led to an anterior chamber paracentesis, which confirmed MPXV as the causative agent. Since first-line antiviral options were not available, therapy was adjusted to oral valaciclovir alongside tapering corticosteroids. Recurrent episodes of keratouveitis were effectively managed with repeated courses of antivirals, corticosteroids, and ocular lubricants, as well as the addition of compounded topical insulin. This case highlights that MPXROD can lead to recurrent keratouveitis even in otherwise healthy individuals. When first-line antivirals are not available, treatment must be individualized, combining antivirals, corticosteroids, supportive care, and adjunctive therapies, such as topical insulin, which can play a crucial role in preserving vision and healing corneal damage in recurrent flares.

## Introduction

Monkeypox is an infectious disease caused by a DNA virus of the genus *Orthopoxvirus* from the family *Poxviridae*, endemic to Central and West Africa. However, an international outbreak was declared by the WHO after 79,411 cases were reported worldwide between January 1 and November 13, 2022, of which 311 were reported in Ecuador [1-2].

The clinical manifestations of monkeypox are similar to those of smallpox and are characterized by skin eruptions, most commonly in the anogenital region, fatigue, and lymphadenopathy [3]. According to a meta-analysis by Gandhi et al., which included 3,239 confirmed monkeypox cases, 755 patients reported ophthalmic manifestations, with the global prevalence of ocular involvement estimated at 9% [4]. Regarding monkeypox-related ocular disease (MPXROD), the most frequently reported manifestations include blepharitis, conjunctivitis, and keratitis [5].

Here, we describe the case of a patient with persistent MPXROD presenting with a range of different manifestations.

## Case presentation

A 28-year-old immunocompetent heterosexual male with an unremarkable medical history and no high-risk sexual behaviors (having maintained one sexual partner in the preceding year) was diagnosed with monkeypox virus (MPXV) infection four months after returning from Spain. During the acute phase, the patient presented with fever and a disseminated rash of umbilicated pustules. About two weeks later, he reported itching, redness, and blurred vision in his right eye; however, ophthalmologic evaluation was delayed due to mandatory isolation.

After completing isolation, the patient sought ophthalmologic evaluation, which revealed reduced visual acuity in the right eye (20/200 uncorrected and 20/70 corrected). Slit-lamp biomicroscopy demonstrated two paracentral dendritic corneal ulcers, pigmented keratic precipitates, anterior chamber cell reaction, and ocular hypertension, while the fundus examination showed no abnormalities. Herpetic keratitis was initially suspected, so treatment was initiated with oral and topical acyclovir, in combination with prednisolone, timolol, dorzolamide, brimonidine, and sodium hyaluronate. The corneal ulcers healed with this regimen, although a residual stromal leukoma remained, partially affecting the visual axis.

**Figure 1:**
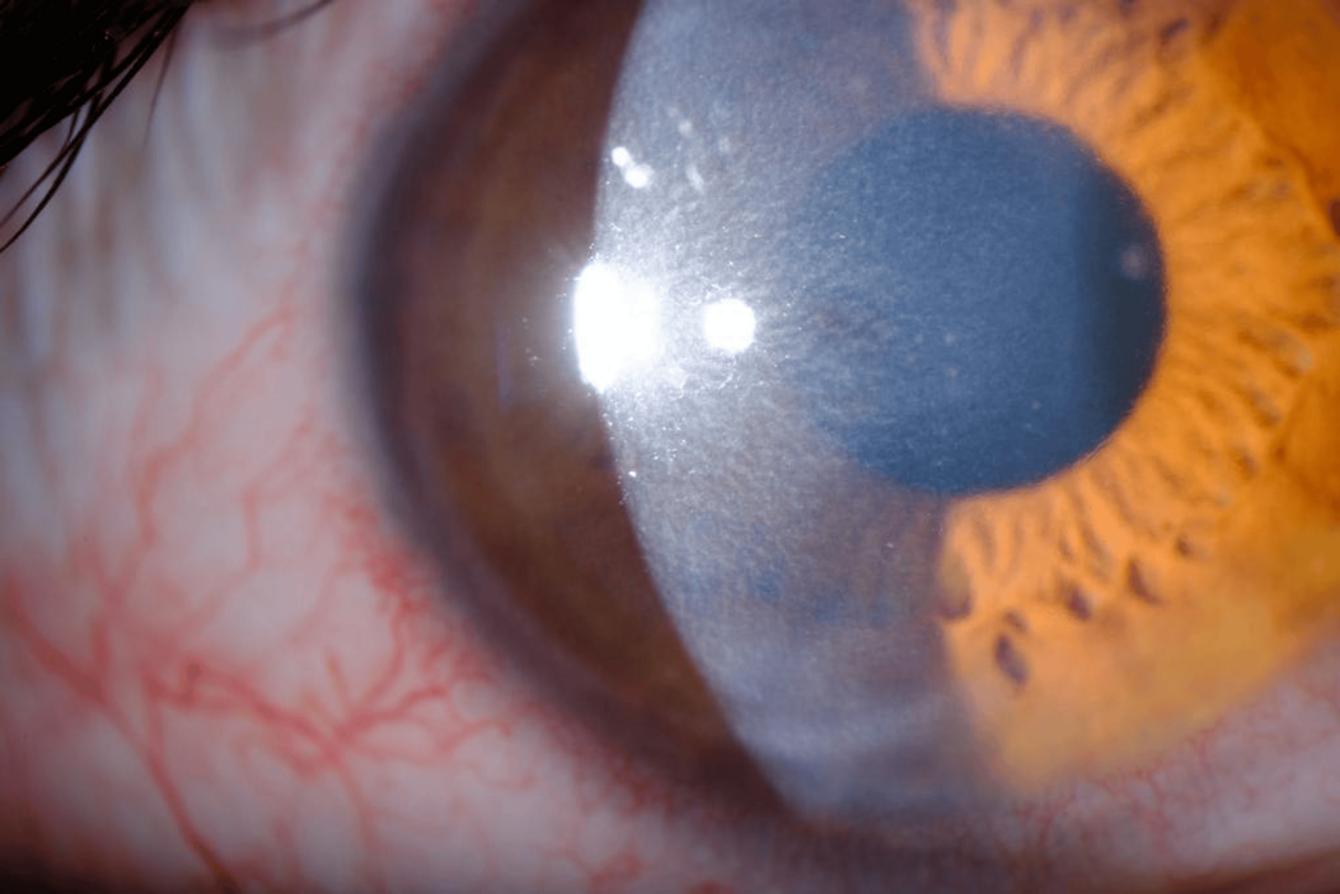
Right eye with paracentral stromal and epithelial edema (three months after the initial episode)

**Figure 2:**
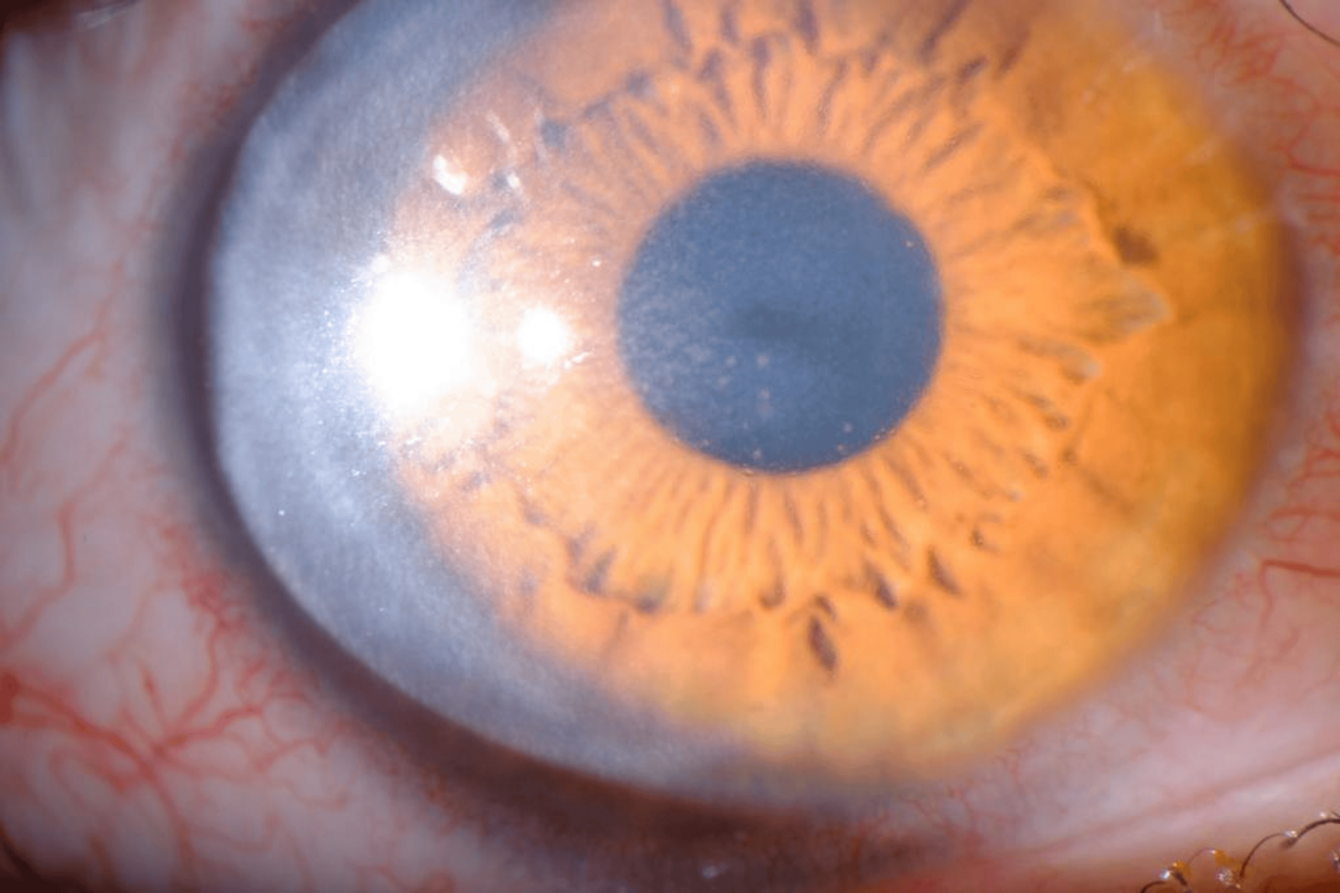
Right eye with pigmented keratic precipitates (three months after the initial episode)

During follow-up, the patient experienced another inflammatory flare in the same eye, marked by pain, redness, blurred vision, corneal edema, and keratic precipitates, findings suggestive of keratouveitis (Figures 1-2). As these features were inconsistent with classic herpetic keratitis and given the background of recent MPXV infection, an anterior chamber paracentesis of the right eye was performed for diagnostic clarification. PCR testing of the aqueous humor was negative for herpes simplex virus (HSV)-1 and HSV-2 but positive for MPXV, confirming disciform keratitis secondary to monkeypox infection.

As first-line antiviral therapy with tecovirimat was unavailable in Ecuador, and neither cidofovir nor trifluridine could be accessed, management was switched to oral valaciclovir, followed by the introduction of tapering topical and systemic corticosteroids. A complementary infectious workup, including Fourth-Generation HIV Ag/Ab Combination Test, purified protein derivative (PPD) skin test, and syphilis (venereal disease research laboratory (VDRL) test), was negative. Over the following weeks, the keratitis improved significantly, with reduced stromal opacity and visual recovery to 20/100 uncorrected and 20/20 with correction in both eyes.

Despite this improvement, the patient experienced recurrent episodes of keratouveitis in the right eye over the following months, each requiring the reintroduction of oral and/or topical antivirals, topical corticosteroids, and lubricants, with consistently favorable responses. It is also worth noting the marked clinical improvement observed after the addition of topical insulin (1 IU/mL) four times daily to the therapeutic regimen. This compounded preparation has shown significant benefits in corneal healing by promoting cellular migration and reducing oxidative stress.

The patient continues under regular ophthalmologic follow-up. Although he has had occasional relapses, his overall progress has been positive, with good preservation of vision and no lasting complications that interfere with his daily life.

## Discussion

Most cases of MPXROD respond well to supportive care alone, although some cases may progress to severe or sight-threatening disease. In this patient, the disease took a more persistent course, initially manifesting as what can be described as a disciform keratitis and later progressing with recurrent episodes of keratouveitis. Only a few cases of stromal involvement in MPXROD have been reported, most showing good outcomes with tecovirimat, but evidence remains limited when this drug is not available [6].

Although monkeypox is self-limiting in most cases, tecovirimat is recommended in instances of severe ocular disease, corneal affection, or persistent infection. While there are no clinical trials directly comparing tecovirimat with supportive care, several small-scale studies have reported favorable responses to tecovirimat [6-8]. Other treatments that have been used against monkeypox-related severe ocular disease (MPSROD) include cidofovir, which has activity against smallpox viruses, and topical trifluridine, which is recommended by the CDC [3-8].

In our patient, although tecovirimat is the recommended first-line treatment in similar cases, this medication was unfortunately unavailable in Ecuador, as were cidofovir and trifluridine; therefore, an alternative approach was required. Consequently, the patient was treated with antivirals typically used for viral keratitis (herpetic keratitis), such as valaciclovir, which is not known to have direct activity against MPXV. Considering the patient’s favorable response to the combination of antivirals and corticosteroids during the initial episode, and the limited therapeutic alternatives available, treatment with valaciclovir followed by corticosteroids was initiated. Although a previous report described prolonged monkeypox infection after corticosteroid use, in that case, the patient had received corticosteroids before starting antiviral therapy, unlike in our case, where antivirals were administered first [9]. Although the use of steroids is generally limited in viral keratitis, prednisolone was administered to address the development of disciform keratitis and keratouveitis relapses by reducing the immune-mediated component of the disease, resulting in a favorable response. Some studies suggest that when used in conjunction with antiviral or antimicrobial therapy, steroids can help control infections. In conditions such as keratitis, endotheliitis, trabeculitis, and uveitis, topical corticosteroids remain the first-line option to ease the intense inflammatory response typically triggered by herpes simplex virus [10-11]. To our knowledge, only one similar case of disciform keratitis associated with monkeypox has been reported in the literature, described by Alsarhani et al. in 2023, where the patient was successfully treated with tecovirimat followed by prednisolone acetate; in contrast, our patient achieved favorable outcomes without access to tecovirimat [7].

Due to recurrence of the infection and the lack of other available options, a topical ophthalmic insulin regimen was initiated for the treatment of keratouveitis, resulting in a good therapeutic response. Regular topical insulin is thought to support corneal repair through the healing of epithelial defects, a process essential for alleviating pain and restoring visual function in persistent ocular conditions [12]. Although no randomized clinical trials are currently available, a 2017 study by Wang et al. involving six patients with refractory neurotrophic corneal ulcers, including cases secondary to herpetic keratitis, reported favorable outcomes with the use of topical insulin drops at a concentration of 1 unit/mL. Although the exact mechanism is not fully understood, the presence of insulin receptors on the cornea points to a role in supporting wound healing and re-epithelialization, with reported recovery times ranging from seven to 25 days after treatment [13-14].

This case illustrates the complexities of managing MPXROD in resource-limited settings and emphasizes the potential utility of topical insulin as an accessible adjunct to support the healing of epithelial corneal lesions.

## Conclusions

MPXROD can cause repeated episodes of keratitis and keratouveitis, even in otherwise healthy individuals. When first-line antivirals, such as tecovirimat, are not accessible, carefully tailored regimens combining oral antivirals, corticosteroids, and ocular lubricants can achieve good results. Adding compounded topical insulin may further aid corneal healing and support the recovery of visual function.

This case highlights the importance of considering MPXV when evaluating patients with unusual ocular inflammation following monkeypox infection. It also demonstrates that thoughtful, personalized management combined with regular follow-up can maintain vision even through intermittent flares. In addition, readily available adjunctive treatments like topical insulin may provide valuable support in resource-limited settings and warrant future research.
